# The application of machine learning in predicting post-cardiac surgery acute kidney injury in pediatric patients: a systematic review

**DOI:** 10.3389/fped.2025.1581578

**Published:** 2025-08-12

**Authors:** Sxe Chang Cheong, Shing Lok So, Alexander Lal, Jan Coveliers-Munzi

**Affiliations:** ^1^ School of Medicine and Population Health, University of Sheffield, Sheffield, United Kingdom; ^2^ School of Health and Medical Sciences, St. George's, University of London, London, United Kingdom

**Keywords:** machine learning, acute kidney injury, cardiac surgery, pediatric patients, risk prediction, dynamic modeling

## Abstract

**Introduction:**

Acute kidney injury (AKI) frequently complicates pediatric cardiac surgery with high incidence and outcomes. Conventional markers (KDIGO criteria) often fall short for pediatric patients undergoing cardiac surgery. Emerging machine learning models offer improved early detection and risk stratification. This review evaluates ML models' feasibility, performance, and generalizability in predicting pediatric AKI.

**Method:**

This systematic review adheres to PRISMA-DTA guidelines. Search was conducted on PubMed and Medline (Ovid/Embase) on March 24, 2024, using PICOTS-based keywords. Titles, abstracts, and full texts were screened for eligibility. Data on study characteristics and best-performing ML models' AUROC, sensitivity, and specificity were extracted. PROBAST evaluated risk of bias and applicability comprehensively. A narrative synthesis approach was employed to summarize findings due to heterogeneity in study designs and outcome measures.

**Results:**

Nine unique studies were identified and included, eight focused on post-cardiac surgery, and one on both PICU admissions and post-cardiac surgery patients. PROBAST demonstrated high risk of bias and low applicability amongst the studies, with notably limited external validation.

**Conclusion:**

While ML models predicting AKI in post-cardiac surgery pediatric patients show promising discriminatory ability with prediction lead times up to two days, outperforming traditional biomarkers and KDIGO criteria, findings must be interpreted cautiously. High risk of bias across studies, particularly lack of external validation, substantially limits evidence strength and clinical applicability. Variations in study design, patient populations, and outcome definitions complicate direct comparisons. Robust external validation through multicenter cohorts using standardized guidelines is essential before clinical implementation. Current evidence, though promising, is insufficient for widespread adoption without addressing these methodological limitations.

**Systematic Review Registration:**

PROSPERO CRD420250604781.

## Introduction

Acute kidney injury (AKI) is a common complication in children following cardiac surgery, affecting 44% to 60% of patients and contributing to increase adverse events such as chronic kidney disease (CKD), extended hospital stays, hemodynamic instability, and increased mortality and morbidity (1–7).

At present, an increase in serum creatinine (sCr) of at least 0.3 mg/dl within 48 h or 50% from baseline within seven days and/or a urine output of less than 0.5 ml/kg/h within six hours, as defined by the Modified Kidney Disease: Improving Global Outcomes' (KDIGO) criteria, remains widely used in clinical practice (8, 9), but might have limited applicability in more critically ill younger population (10).

In the context of pediatric cardiac surgery with cardiopulmonary bypass (CPB), significant stress and inflammatory response is imposed on multiple organ systems, resulting in a complex interplay that is not yet fully understood (11–14). In pediatric patients, immature organ systems render them inherently more vulnerable and sensitive to surgical insults; hence, minor intraoperative fluctuations can lead to significant adverse effects compared to adults (15). Furthermore, unique surgical risk factors, such as prolonged CPB time, extended cross-clamp time, and factors that cause hypoperfusion, kidney injury and inflammation, may not be adequately captured by the KDIGO criteria (16). In the pediatric population, factors such as young age, fluid overload, and the duration of CPB are well known as major risk factors for post-cardiac surgery acute kidney injury (CS-AKI) (4), whereas in adults, more prevalent co-existing comorbidities like hypertension and diabetes, along with variations in CPB techniques, play a larger role in the prognosis of AKI post-surgery (17). Ultimately, these differences in physiology, risk factors, and intraoperative variables between adults and children necessitate the development of a more dependable clinical prediction model.

Following initiatives such as AWAKEN and AWARE, to redefine and address the lack of consensus of AKI in the neonatal and pediatric population (18, 19). Identifying clinically significant AKI, that is, kidney injury which not only meets biochemical thresholds but also demonstrably impacts patient outcomes, particularly morbidity and mortality, may serve as a better alternative to the current criteria (20). As such, a rise in serum creatinine or a decrease in urine output does not always accurately indicate AKI, or necessitate treatment. Moreover, given the often-delayed presentation of AKI, a clinical prediction model could be more appropriate, as preemptive treatment can lead to significantly improved prognoses in the pediatric population (21).

Promising serum and urinary biomarkers, such as neutrophil gelatinase-associated lipocalin (NGAL), have been correlated with AKI severity and can rise within 2–6 h post-injury, significantly earlier than creatinine (22), which typically rises after 24–36 h. Other biomarkers, including kidney injury molecule-1 (KIM-1), cystatin C (23), interleukin-18 (24), and L-FABP, have an earlier detection window of 4–6 h (25, 26). Although these markers exhibit high (Area Under the Receiver Operating Characteristic Curve) AUROC values, their clinical implementation remains limited due to issues with non-specificity, high cost, lack of validation, and insufficient consideration of significant age-dependent risk factors (27, 28).

Clinical prediction models that incorporate machine learning (ML) frameworks show great promise in analyzing and handling complex relationships between numerous factors involved in AKI development (29). By enabling clinicians to stratify patients by risk and adjust practices accordingly, ML models can offer individualized predictions of AKI by integrating preoperative, intraoperative, and postoperative data (30–36). Models that incorporate real time dynamic data have outperformed KDIGO-based criteria, achieving AUROC values of up to 0.90 in some studies (25). These models provide earlier risk prediction and facilitate timely treatment. This review aims to assess the feasibility, performance, and generalizability of machine learning in predicting pediatric CS-AKI.

Although our search approach focused specifically on patients following cardiac surgery, we included two studies from pediatric cardiothoracic or cardiac ICU environments because they offered important insights into high-risk patient groups, despite not being limited to post-surgical cases.

## Methods

### Search strategy

This systematic review is written and reported according to The Preferred Reporting Items for Systematic Reviews and Meta-Analyses of Diagnostic Test Accuracy Studies (PRISMA-DTA) statement (37). The search was conducted on PubMed (Supplementary Material S1) and Medline via Ovid (including Embase) (Supplementary Material S2), Web of Science (Supplementary Material S3), and Scopus (Supplementary Material S4) on the 24th of March, and was updated on the 27th of June 2025. Grey literature sources such as conference proceedings, clinical trial registries, and institution repositories (StarPlus Library) was searched to identify relevant studies. Initial title and abstract of all retrieved articles were screened for eligibility. Two authors (SLS and AL) independently screened all the articles during initial title/abstract screening and full-text screening phases. A third author (JCM) review and resolve any conflicts that arose during the screening process. Full text was retrieved following initial screening and reviewed against the eligibility criteria.

### Population, index, comparison, outcome, timing, and setting (PICOTS)

This study focuses on pediatric patients (<21 years) admitted to the Pediatric Intensive Care Units (PICU)/ Intensive Care Units (ICU) following cardiac surgery (38).

The primary index is the application of a machine learning framework to predict clinical outcomes. A typical ML framework was defined by three main themes: data collection and processing, model development (including feature selection, optimization, and ML algorithm selection), and model validation and training (covering training and validation sets, cross-validation, and data splitting).

The outcome of interest is the development of AKI of any stage, with a specific focus on severe AKI occurring post cardiac surgery during the ICU or PICU admission period. The prediction and assessment of these outcomes are conducted during the immediate postoperative phase, and the study is set in ICU and PICU in healthcare institutions that perform cardiac surgery on pediatric patients.

Studies were excluded if they did not meet the PICOTS framework, including those that involved adults (>21 years), did not utilize an ML framework, did not mention pediatric cardiac surgery patients, were not published in English, failed to report diagnostic accuracy metrics of interest, had incomplete full texts, or were conference abstracts, review articles, or editorials.

### Data extraction and narrative synthesis

The same multi-author approach was maintained throughout data extraction with the same authors (SLS and AL) working independently and third author (CSC) resolving discrepancies. Basic study and population characteristic, inclusion, and exclusion criteria, primary outcome, and objective were compiled and presented in tabular format. Only the best-performing ML model's metrics, AUROC, sensitivity, and specificity, related to this review's objective were extracted. For studies that conducted external validation, both internal and external results were reported. Data were recorded as means with standard deviations or 95% confidence intervals, as provided. A qualitative review analyzed study limitations and author recommendations, while descriptive analysis summarized study characteristics.

### Risk of bias assessment

The Prediction model study of Risk of Bias Assessment Tool (PROBAST) was used to assess the risk of bias and applicability of the models, covering four domains (Participants, predictors, outcomes, analysis) (39). All authors conducted the assessment independently; responses were reviewed at the end to address any disagreements.

## Results

**Figure 1:**
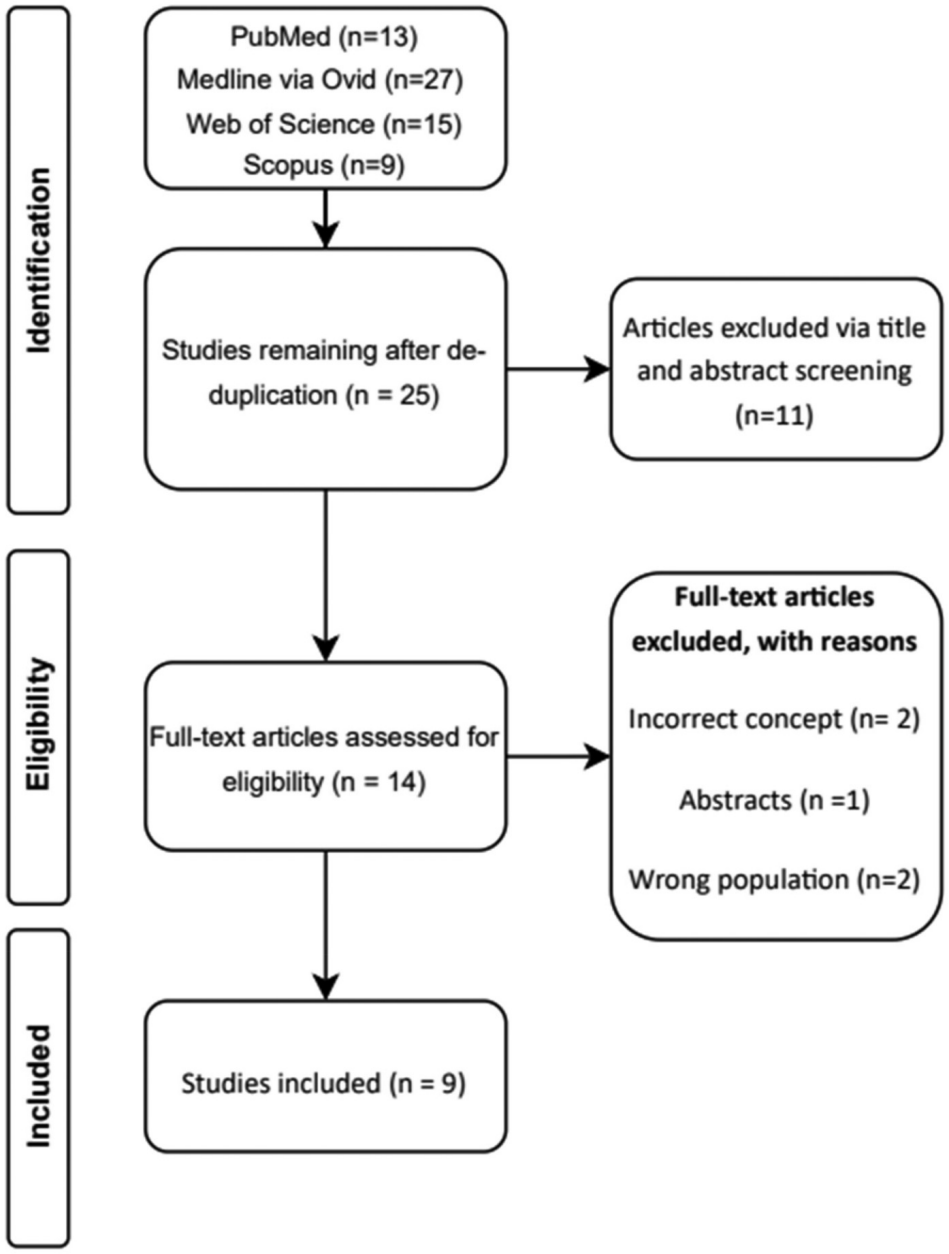
PRISMA flow diagram.

A total of 48 studies were identified via specified databases. After de-duplication, 25 unique studies remain and was screened. Initial title and abstract screening excluded 11 articles. Full text was retrieved for 14 studies, and five full-text articles were excluded with reasons provided (Figure 1) (40–44). Nine unique studies were identified and included in this review, with eight including post-cardiac surgery patients, and one on both cardiothoracic and ICU units (30–32, 34–36, 45–47) (Table 1).

**Table 1:** Summary of studies' objectives, primary outcomes, and eligibility criteria.

				Eligibility criteria				
Study (Year) (reference)	Country	Study design	Collection date	Inclusion	Exclusion	Primary outcome; additional outcome	Population size	Demographics [Median age (IQR), Female %]
Dong. et al.^a^ (25)	US and UK	Multicenter retrospective observational study	2003–2019	Pediatric patients from PICU and cardiothoracic intensive care units (CTICU) from (1 months to 21 years)	Neonates, AKI within 12 h, ICU stay <24 h	Prediction of moderate to severe AKI up to 48 h–6 h in advance; Prediction of any stage AKI and RRT use	16,863	Hospital 1: 4 years [0.7, 12.1]; 46%
								Hospital 2: 2 years [0.6, 6.0]; 46%
								Hospital 3: 7 years [3.8, 14.7]; 48%
Fragasso. et al.^a^ (46)	Italy	Retrospective observational study	January 1, 2018–February 29, 2020	Pediatric patients with in pediatric cardiac ICU stay of at least 48 h	Adults' patients (>18 years), with missing data and chronic kidney disease	Prediction of severe AKI (KDIGO stages 2 and 3) after 48 h; Binary AKI prediction (no AKI vs any AKI); Multiclass AKI (Maximum and Mode AKI)	419	164 days (31–999), 46%
Hayward. et al. (31)	UK	single- center Retrospective cohort study	April 2019–April 2021	Cardiac surgery with CPB Pediatric patients	Preexisting AKI, univentricular anatomy, preterm, reoperation, incomplete data, selective cerebral perfusion, deep hypothermic circulatory arrest	Post surgical AKI within 48 h; Association of cumulative time with DO2i < 350 ml/min/m²; AKI risk factors	396	No AKI group: 7 months (3–27)
								AKI group: 4 months (2–11); 42%
Kong. et al. (35)	China	Retrospective cohort study	Jan 2002–Jan 2022	Pediatric patients undergoing aortic arch reconstruction with CPB	Patients with severe preoperative renal injury (eGFR < 30 ml/min/1.73 m² or urine output <0.5 ml/kg/h), sepsis, or incomplete data	Occurrence of AKI post-surgery within 7 days; Risk factors and evaluation of ML models	134	AKI: 2 months (1.0–7.0); 28%
								Non-AKI: 3 months (1.5–9.6); 36%
								Overall Female: 32%
Loomba. et al. (30)	USA	Retrospective single center study	September 2022–March 2023	Neonates with Norwood procedure with available data	Patients requiring ECMO prior to surgery.	AKI post cardiac surgery; Change in creatinine to baseline creatinine ratio	9	Mean 20.5 days (± 32.0); NR
Luo. et al. (47)	China	Multicenter Retrospective cohort study	January 2015–March 2022 (Derivation cohort), January 2016 to December 2021 (External validation cohort)	Pediatric cardiac surgery patients with CPB and SCr measurement pre- and post-surgery	Congenital renal malformation and eGFR (<15 ml/min/1.73 m²)	Development of cardiac surgery-associated acute kidney injury (CS-AKI); CS-AKI (stage 2–3); In-hospital mortality; ICU length of stay; Total postoperative hospital stays	3,863 (Derivation: 3,278, External: 585)	Derivation: 1 year (0.5–4), 47.9%; External Validation: 4 years (1–8), 50.8%
Nagy. et al. (34)	USA	Retrospective cohort study	September 1, 2007–June 31, 2003	Pediatric cardiac surgery patients	Patient with patent ductus arteriosus	Prediction of moderate-to-severe CS-AKI (KDIGO stages 2 or 3) on postoperative day 2 (POD2); Hospital Survival Prediction	402	6 months (2–27), 56%
				<21 years				
Tong. et al. (36)	China	Retrospective cohort study	August 2014–December 2021	Pediatric patients undergoing congenital heart surgery	Patients with missing or unclear data	Major Adverse Postoperative outcomes (Low cardiac output syndrome, pneumonia, renal failure, Deep vein thrombosis); Prediction of ICU/Hospital length of stay	Total: 23,000	11.6 months (5.2–32.7); 46%
							Renal failure: 458 (2.0%)	
Zeng. et al. (32)	China	Retrospective cohort study	2018	Pediatric patient with congenital heart surgery	Patient with insufficient missing data, surgery without CPB, or death	Prediction of postoperative acute kidney injury; Continuous prediction of AKI during stays post-surgery; Prediction of AKI development within a random given time window	3,386	11.9 months (4.5–28.9); 50.6%

^a^ Studies exploring pediatric patients in ICU, did not explicitly mention cardiac surgery.

Most studies were published between 2023 and 2024, with four from China (32, 35, 36, 47), two from the US (30, 34), one from Italy (46), one from the UK (31), and one a collaborative effort between the UK and US (45). All studies were conducted in accordance with the Kidney Disease Improving Global Outcomes (KDIGO) criteria.

Four studies evaluated only a single ML model (30, 34, 45, 46), while one study assessed multiple advanced deep learning models (32) and an additional four studies evaluated more than one model (31, 35, 36, 47) (Table 2). In terms of predictive performance for CS-AKI, random forest emerged as the best performing model in two studies (31, 46), with LightGBM also reported as superior in two studies (34, 36). Logistic regression was identified as the top model in two studies (30, 35), whereas extreme gradient boosting and the time-aware attention-based recurrent neural network were each reported as the best performing model in one study (47) (Table 3).

**Table 2:** Summary of machine learning algorithm used, and key predictors identified.

Author (Year)	Overall incidence of AKI; Moderate-severe incidence (%)	Machine Learning Algorithm used (Best performing model)	Lead time prediction window; primary outcome (criteria)	Input data	inputs (No)	Key Variables identified (Feature selection method)
Dong. et al^a^ (45)	10.6%–19.8%; 3.5%–5.3% (across three hospitals)	Ensemble model (age dependent)	48–6 h before AKI onset (KDIGO stage 2/3)	Electronic health record, vital signs, laboratory, medication, and ventilation parameters	15	Shock index, SpO2, blood urea nitrogen, serum creatinine rate of change, bilirubin, PaCO₂, anion gap, white blood cell count, serum albumin, serum chloride, gentamicin trough, number of vasoactive drugs, number of high nephrotoxic potential drugs, mean airway pressure (for ventilated patients), and time since admission. (Univariate and multivariate model-based selection)
Fragasso. et al^a^ (46)	53.2% (223/419); 30% severe; 18% stage 1; 35% stage 3	Random forest (Ensemble learning method)	Two days (48 h) post ICU admission; Severe AKI (KDIGO 2, 3)	Admission and post-admission data (Demographics, clinical scores, basal serum creatinine, CPB, cross clamp duration) Vital signs, Fluid, Blood gas analysis, Laboratory, Therapeutic	36	Creatinine, CPB duration, Basal creatinine, Platelet count, Lactate dehydrogenase (Important matric plot)
Hayward. et al. (31)	25.8% (102/396)	Random forests (Best performing model); Logistic regression	Within 48 h post-surgery; All stages of AKI (KDIGO)	Pre-operative (e.g demographics) and time series (minute by minute) intraoperative CPB data (e.g oxygen delivery, CPB, inotrope usage)	15–20	Duration under DO2i < 350 ml/min/m² without body surface area (≥ 30 min), patient age (<4.7 or ≥ 6), CPB duration (<71 min or ≥ 86 min), mean arterial blood pressure, and VIS (Multicollinearity analysis of variable importance)
Kong. et al. (35)	50% (Propensity scored/matched)	Extreme gradient boosting, logistic regression (Best performing model), light gradient boosting machine, GaussianNB, multilayer perceptron, and support vector machine.	Within 7 days post-surgery; Any stage AKI (KDIGO and RIFLE)	Pre-operative, intraoperative and postoperative surgical data.	15–16	weight, eGFR, cyanosis, PDA, newborn status, and duration of renal ischemia. (Univariate logistic regression and LASSO regression)
Loomba. et al. (30)	22% (2/9)	LASSO logistic regression	2–4 days post-surgery; All stages of AKI (KDIGO)	Hemodynamic data collected every 5 s and linked to serum creatinine measurements collected every 24 h	∼18 variables	Net fluid balance and renal oxygen extraction (LASSO)
Luo. et al. (47)	8.7%–17.2%; 4.4%–6.3% (Across multiple samples)	XGBoost (Best performing), K-nearest neighbor, Naive Bayes, Support Vector Machines, Random Forest, Neural Networks	Within two to seven days post cardiac surgery; All stages of AKI (KDIGO)	Preoperative data and intraoperative data	Preoperative only model: 25, Combined (Preoperative and intraoperative): 20	Baseline serum creatinine. perfusion time, body length, operation time, and intraoperative blood loss. (Least Absolute Shrinkage and Selection Operator; LASSO, Boruto algorithm, random-recursive feature elimination, and random forest-filtering)
Nagy. et al. (34)	13.7% (55/291 for moderate–severe AKI)	Light GBM with SHAP	Postoperative day 2; AKI stages 2 or 3 (KDIGO)	Demographic, preoperative, intraoperative, and immediate postoperative (POD0) clinical and laboratory data. POD0 are obtained within 60 min upon arrival at ICU	34	Preoperative serum creatinine, Total duration of surgery. POD0 serum pH, POD0 lactate, Cardiopulmonary bypass duration, POD0 vasoactive inotropic score, Sex, POD0 hematocrit, Preoperative weight, POD0 serum creatinine (SHAP)
Tong. et al. (36)	2% (458/23,000; renal failure incidence)	LightGBM (Best performing model), Logistic regression, Support vector machine, Random Forest, CatBoost	Post cardiac surgery to discharge; All stages of renal failure (KDIGO)	Combined clinical and laboratory data	39	Mechanical ventilation time, STAT score, and operative time (SHAP)
Zeng. et al. (32)	9.8% (331/3,386)	Time aware attention based Recurrent Neural Network (RNN)- Long Short-Term Memory network (Best performing);	Perspective 1: 168 h to 6 h (within 7 days); All stages of AKI (KDIGO)	Static and time-series features with sampling frequency (Intraoperative vital sign, laboratory tests, and blood gas)	83	Varies with different perspectives (common key predictors): Autologous blood transfusion during cardiopulmonary bypass (CPB), mechanical ventilation time, CPB time, cross-clamp time, age, postoperative length of stay (LOS), packed red blood cell (PRBC) transfusion during surgery, weight, total anomalous pulmonary venous connection repair, and postoperative hospital stay. (Shapley Additive Explanations; SHAP)
		Support vector machine and logistic regression;	Perspective 2: Entire postoperative ICU stay post-surgery; All stages of AKI (KDIGO)			
		Plain Recurrent neural network;				
		Gated recurrent units (GRUs);				
		Attention based models – Dipole and RETAIN				
			Perspective 3: Random time point (6, 12, or more); All stages of AKI (KDIGO)			

^a^ Studies exploring pediatric patients in ICU, did not explicitly mention cardiac surgery.

**Table 3:** Summary of performance metric of machine learning models.

Author (Year)	ML model; Stage of AKI (Criteria)	Accuracy	Area under the curve	Sensitivity or recall	Specificity	PPV/Precision	NPV	F1-score; Brier score loss (95% CI)
		(95% CI)	(95% CI)	(95% CI)	(95% CI)	(95% CI)	(95% CI)	
Dong. et al^a^ (45)	Ensemble ML; Stages 2/3 and All stages of AKI (KDIGO)	NR	Any stage AKI (Stages 1, 2 or 3): 0.83 to 0.89	Any stage AKI (Stages 1, 2 or 3): 0.41	NR	ML model	NR	NR
						Any stage AKI: 0.47		
			Moderate to Severe AKI (stages 2 or 3): 0.89	Moderate to Severe AKI (stages 2 or 3): 0.58		AKI stage 2/3: 0.22		
Fragasso. et al^a^ (46)	Random forest; Stages 2/3 and Binary AKI/ All stages of AKI (KDIGO)	NR	Severe: 0.99 (0.98–1)	Severe: 0.74	Severe: 0.99	Severe: 0.94	Severe: 0.97	NR
				Binary: 0.71	Binary: 0.98	Severe: 0.92	Severe: 0.92	
			Binary: 0.93 (0.92–0.94)					
Hayward. et al. (31)	Random forests (500 decision trees); All stages AKI (KDIGO)	0.66	0.67	NR	NR	NR	NR	NR
Kong. et al. (35)	Logistic Regression; All stages AKI (KDIGO and RIFLE)	0.821	0.889	0.832	0.816	NR	NR	NR; 0.129
Loomba. et al. (30)	LASSO logistic regression; All stages AKI (KDIGO)	0.89	0.73	0.96	0.90	0.12	0.99	NR
Luo. et al. (47)	Extreme Gradient Boosting model (Combined model with 27 variables); All stages AKI (KDIGO)	NR	Derivation: 0.912 (0.899–0.924)	Derivation: 0.95 (Cut off value: 0.099)	Derivation: 0.58 (Cut off value: 0.099)	Derivation: 0.32 (Cut off value: 0.099)	Derivation: 0.98 (Cut off value: 0.099)	Derivation: 0.085; NR
			External Validation: 0.889 (0.8444–0.920)					External: 0.060; NR
				0.60 (Cut off value: 0.374)				
					0.95 (Cut off value: 0.374)	0.72 (Cut off value: 0.374)	0.92 (Cut off value: 0.374)	
				External Validation: 0.80 (Cut off value: 0.099)				
					External Validation: 0.81 (Cut off value: 0.099)	External Validation: 0.28 (Cut off value: 0.099)	External Validation: 0.98 (Cut off value: 0.099)	
				0.28 (Cut off value: 0.374)				
					0.99 (Cut off value: 0.374)	0.67 (Cut off value: 0.374)	0.93 (Cut off value: 0.374)	
Nagy. et al. (34)	Light GBM; Stages 2/3 (KDIGO)	0.91 (0.82–1.00)	0.88 (0.72–1.00)	0.63 (0.32–0.96)	NR	0.92 (0.70–1.00)	NR	0.73 (0.46–0.99); 0.09 (0.00–0.17)
Tong. et al. (36)	LightGBM; All stages of AKI (KDIGO)	0.871	0.963 (0.947–0.979)	0.870	0.958	NR	NR	NR
Zeng. et al. (32)	Time aware attention based recurrent neural network, LSTM network (Perspective 2 at 24 h); All stages AKI (KDIGO)	0.832 (0.830–0.833)	0.908 (0.907–0.909)	0.911 (0.904–0.918)	NR	NR	NR	NR; 0.127 (0.126–0.128)

^a^ Studies exploring pediatric patients in ICU, did not explicitly mention cardiac surgery.

### Key predictors

Recurring risk factors identified across studies using various feature selection methods to optimize AKI prediction can be stratified into several categories. Renal function parameters—including baseline, postoperative day one, and the rate of change of SCr, blood urea nitrogen, and urine output—were found to be particularly significant. Intraoperative metrics such as CPB duration, surgery duration, perfusion time, and intraoperative blood loss also play a key role. Patient demographics, notably body weight and age, alongside respiratory and hemodynamic measures like mechanical ventilation time, mean arterial blood pressure, shock index, and mean airway pressure, correlate with the severity of AKI. Additionally, laboratory and biomarker data, including lactate dehydrogenase, white blood cell count, bilirubin, serum albumin, serum chloride, anion gap, and PaCO₂, provide indirect indicators of AKI risk (Table 3).

### ML frameworks

Various machine learning (ML) pipelines can be applied to data analysis depending on the research objectives and the nature of the dataset. Certain ML techniques perform better when applied to appropriately preprocessed data. In many studies, multiple models are evaluated to identify the best-performing one based on interpretability, predictive accuracy, or computational efficiency (48). The models used can generally be categorized into linear models, ensemble learning methods, tree-based methods, and neural networks or deep learning approaches.

Linear models, such as logistic regression, are typically supervised and assume a linear relationship between input features and the outcome. Ensemble learning methods build upon multiple decision trees and combine the outputs from different subsets of data to produce more robust and unbiased predictions. Tree-based methods, like decision trees, classify data by recursively splitting it into subsets based on feature thresholds. Lastly, deep learning involves complex architectures with multiple interconnected layers of nodes, capable of capturing intricate, non-linear patterns in the data (49).

### Ensemble and tree-based models

Fragasso et al. (2023) enrolled 419 patients and reported an AKI incidence of 53.2% (223/419) overall: specifically, 30% (125/419) at stage 1, 18% (75/419) at stage 2, and 35% (148/419) at stage 3. A random forest ML model was implemented under four different definitions: (1) no AKI vs. AKI, (2) no/mild AKI vs. severe AKI, (3) the maximum AKI stage, and (4) the most frequent AKI stage. Each approach achieved respective AUCs of 0.93, 0.99, 0.92, and 0.95 at 48 h (46).

Hayward et al. (2023) analyzed 396 pediatric surgery patients on CPB. They found that maintaining an oxygen delivery level above 350 ml/min/m³ was crucial; patients below this threshold had a significantly higher risk of postoperative AKI. Using a random forest method, the model reached an AUC of 0.67. The overall AKI incidence was 25.8% (102/396), with diminished urine output occurring in more patients than elevated creatinine (18.9% vs. 6.9%, respectively, among the 102 cases) (31).

Meanwhile, Tong et al. (2024) analyzed 23,000 children undergoing congenital heart surgery, where 2% (458/23,000) developed renal failure. Their Light GBM framework accurately forecasted adverse outcomes with an AUC of 0.963 and a sensitivity of 87%. Including both clinical and laboratory features led to more accurate predictions than using clinical parameters alone (36).

In a smaller retrospective study, Nagy et al. (2024) focused on 402 pediatric patients following cardiac surgery. LightGBM effectively differentiated no/mild AKI from moderate/severe AKI by postoperative day 2, delivering an AUC of 0.88 and a sensitivity of 0.63—substantially higher than the 0.70 AUC achieved by the cardiac renal angina index (cRAI). Among these 402 children, 13.7% (55/402) experienced moderate or severe AKI (34).

Dong et al. (2021) developed a single predictive model using data from 16,863 patients across three hospitals. The study reported that all stages of AKI occurred in approximately 10.6% to 19.8% of patients, while moderate to severe AKI was observed in 3.5%–5.3% of cases. The model achieved an AUROC of 0.89 and was able to identify stage 2/3 AKI a median of 30 h (within a range of 24–48 h) before its onset. It successfully identified 40% of all AKI episodes, had a 58% sensitivity for stage 2/3 AKI, and detected 70% of cases that eventually required renal replacement therapy. However, the model's performance was reduced in the UK hospital, likely due to a smaller, imbalanced dataset and cohort size. Every six hours, the ensemble assigns age-adjusted weights to each predictor and sums them to generate an overall AKI risk probability, facilitating timely monitoring during critical shifts (45).

Luo et al. (2023) evaluated 3863 pediatric patients using the extreme gradient boosting model, finding an overall AKI incidence between 8.7% and 17.2% and a moderate-to-severe AKI rate of 4.4%–6.3% across internal and external validations. They developed six models under two scenarios: one using only preoperative data and another that combined preoperative with intraoperative data. The XGBoost model proved most effective, and including intraoperative data improved performance in both cohorts, with the AUROC increasing from 0.890–0.912 in the derivation cohort and from 0.857–0.889 in the external validation cohort (47).

### Linear model

Kong et al. (2023) studied 134 propensity-matched children having aortic arch reconstruction and found a 50% rate of AKI. Renal ischemia time was the strongest predictor (OR 1.169, 95% CI 1.092–1.251), and most AKI cases involved preoperative cyanosis. Their logistic regression model, outperforming other algorithms, yielded an AUC of 0.89 in training and 0.84 in testing, with AKI assessed within a seven-day window. They also built a nomogram projecting AKI risk one year later (35).

Loomba et al. (2024) examined nine neonates with univentricular hearts undergoing the Norwood procedure, where 22% developed AKI. Key drivers were net fluid balance and oxygen extraction, yielding an AUC of 0.73. By continuously tracking hemodynamics through T3 software, they concluded that pressures (blood or renal perfusion) had little bearing on AKI onset (30).

### Neural network

In a cohort of 3,386 pediatric patients, Zeng et al. (2023) tested seven models in three different analyses using data captured 24 h post-surgery, where AKI occurred in 9.8% of cases (331/3,386). These models offered lead times from 6 h up to 7 days. Notably, the Time-Aware Attention-Based LSTM outperformed all other methods across every analysis, achieving an AUC of 0.908 (95% CI 0.907–0.909). Moreover, results were strongest when using data collected 24 h after surgery. The approach handles time-based clinical data in a recurrent architecture that captures sequential measurements, highlighting key shifts over time. An attention mechanism emphasizes the most critical segments for predicting AKI, and the resulting probabilities are fine-tuned through Platt scaling and isotonic regression (32).

### Risk of concerns -PROBAST

**Figure 2:**
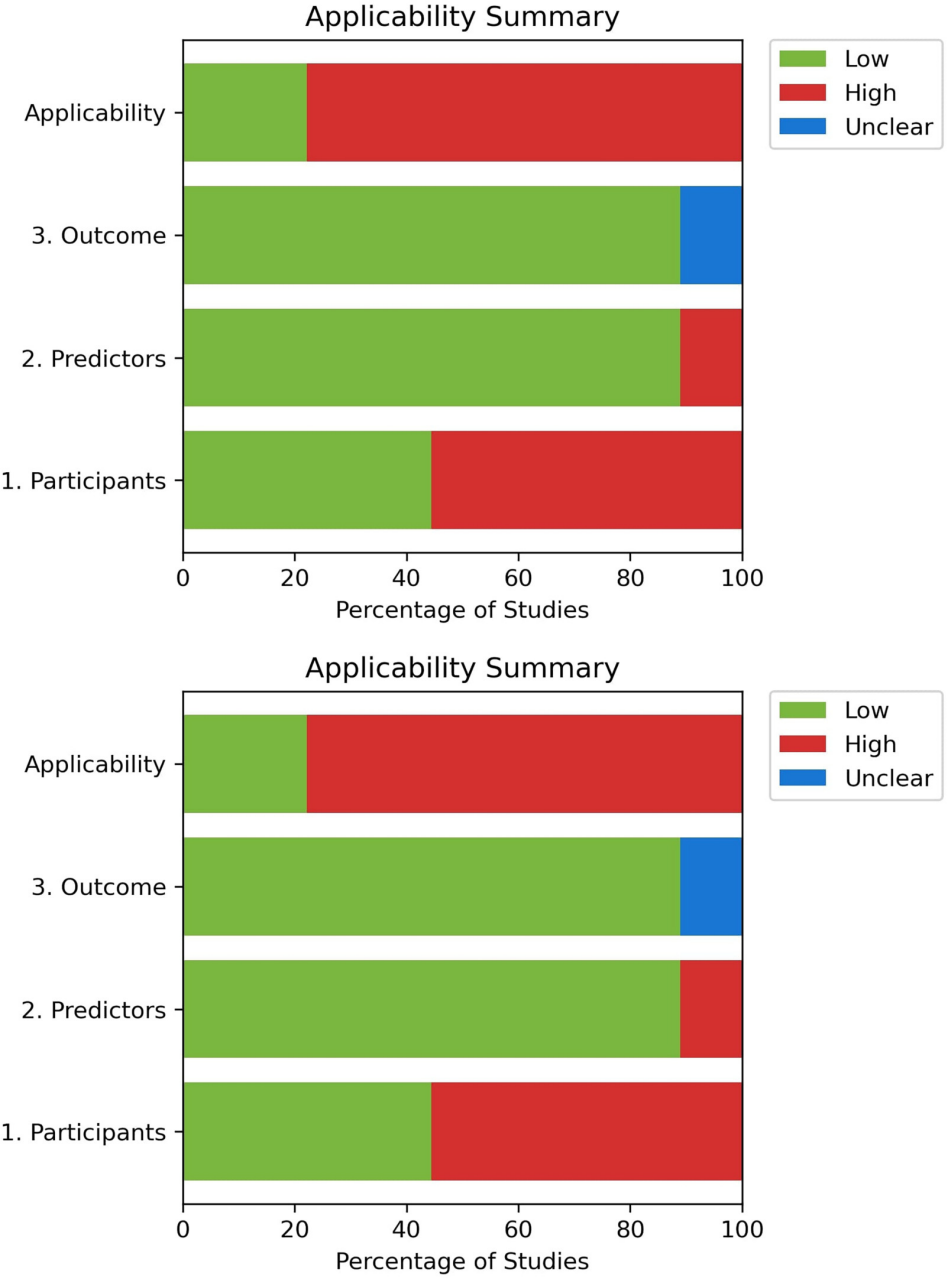
PROBAST risk of bias summary and applicability of summary.

Across the nine included studies, most demonstrated high risk of bias in participant selection, predictor assessment, outcome measurement, and analysis domain due to the lack of external validation of their predictive models, limiting confidence in generalizability (30–32, 34–36, 46). Sample sizes were often small, further restricting robust analysis and replication (Figure 2). Moreover, one study included ICU populations raising concerns about its applicability in predicting CS-AKI (46).

### Limitations of ML reported by authors

Each study faced unique challenges in the diagnosis of AKI (see Supplementary Table S1). Common limitations reported across studies were retrospective (*n* = 9) (30–32, 35, 36, 45–47, 47) and single-center designs (*n* = 7) (30–32, 34–36, 46). Small or homogenous cohorts (*n* = 4) (30, 34, 35, 46) limits the generalizability of the model. Missing or limited variables, such as exclusion of key predictors (e.g., urine output data, nephrotoxic medications, surgical risk scores) (*n* = 5) (30, 32, 45–47), and reliance on estimated baseline measures or incomplete intraoperative details (*n* = 2) (34, 36). Advanced ML methods, while good at handling complex data, could introduce “black box” models that are less interpretable (*n* = 3) (32, 36, 47), limiting clinical integration. Lack of external validation raises concerns about the robustness and generalizability of the predictive model (*n* = 6) (30, 32, 35, 36, 46, 47).

## Discussion

The high heterogeneity across each study, due to differences in study design, ML frameworks, populations, and input variables makes direct comparison via meta-analysis challenging. The use of ML models to predict AKI development in post cardiac surgery demonstrated strong discriminatory power. Several retrospective (30–32, 34–36, 45–47) and in a prospective study reported AUC values in the range of 0.80–0.90, often outperforming conventional and novel investigative tools, urine and serum biomarkers (32, 34–36, 45–47). These models can provide early warnings up to 24–48 h prior to conventional diagnostic tools thereby facilitating timely prophylactic interventions and potentially more efficient resource allocation.

Many studies in this review utilized random forests and gradient boosting, which generally outperformed other algorithms. The ensemble approaches offers greater interpretability compared to deep learning models, which are often criticized as “black boxes” due to the lack of transparency in its decision-making process (32, 36, 47). However, when it comes to unstructured data, such as analyzing continuous time-series data, unsupervised deep learning techniques could potentially outperform classical supervised framework.

The benefits of using ML over traditional statistical methods are its ability to detect complex interactions among many parameters. Furthermore, several studies were large multi-center investigations with external validation, which indicated potential broad applicability and integration into existing clinical frameworks. With larger population sizes and higher-quality data, predictions are likely to be more accurate and applicable. Most studies included feature identification and interpretability tools such as SHAP (32, 34, 36), LASSO (30, 35, 47), and other forms of feature reduction techniques (31, 35, 45–47) to enhance transparency and provide clinicians with insights into its decision-making process.

The potential weaknesses of ML, as with any approach, include the crucial dependency on the data fed into the framework. Single-center data can restrict generalizability, as it often reflects specific on-site practices and protocols (such as inotropic medications or fluid transfusion protocols), demographics (such as ethnicity), and epidemiology. This can encourage overfitting, making the model less suitable when applied to other cohorts, as noted by several studies (45). While the use of time-series or temporal measurements, where data is collected frequently, can facilitate more precise monitoring of the inherently labile pediatric physiology, missing data may pose issues that limit the model's robustness. Lastly, many of the studies conducted were offline retrospective analyses (30–32, 34–36, 45–47)—a necessary first step that should ideally be followed by prospective studies to provide insights into real-time clinical workflows.

Timing plays an important role in determining the accuracy of the model, several studies found that a short lead time or prediction window, as early as 6–48 h pre surgery or post-surgery produces greater diagnostic accuracy compared to longer periods (32, 45).

Preoperatively, low baseline kidney function, younger age, and cyanosis or ventricular anomaly is found to be major contributor to AKI risk (47, 50). Intraoperative risk factors specific to cardiac surgery patients were prolonged CPB and renal hypoperfusion, longer operation time, and greater surgical complexity were linked with higher risk of AKI development (31, 35). As for post-operative risk factors, longer mechanical ventilation and higher vasoactive inotrope use were correlated with AKI risk (36).

Several studies explored the application of machine learning in predicting AKI in PICU/ICU patients, achieving high discriminatory power. Hu et al. (2023) studied 957 critically ill PICU patients across four hospitals (for a total evaluated population of 1,843), with an AKI incidence of 24.6% (449/1,843). Compared to non-AKI patients, those with AKI were more likely to require renal replacement therapy, had extended PICU stays, a higher rate of serious complications, and increased mortality. Of 11 ML algorithms tested, the random forest model performed best when focusing on eight key features, yielding AUCs of 0.929 (internal validation) and 0.910 (external testing). It predicted AKI more accurately on day 1 (AUC 0.977) than on days 2–7 (AUC 0.927), and its predicted risk closely aligned with adverse clinical outcomes (50).

Xu et al. (2024) investigated eight distinct predictive algorithms for acute kidney injury (AKI) and acute kidney disease (AKD) in a cohort of 1,685 hospitalized pediatric patients, reporting an AKI incidence of 14.90% (251/1,685) and an AKD incidence of 16.26% (274/1,685). Among the evaluated models, Light Gradient Boosting Machine (LightGBM) achieved the highest area under the curve (AUC) at 0.813 for AKI, outperforming Naïve Bayes (AUC 0.791) and Random Forest (AUC 0.784). Future studies should explore whether the use of generalized AKI prediction models can achieve high accuracy and be applicable in post-CS-AKI patients (33).

### Serum and urinary NGAL, serum cystatin C, urine IL-18, and urine L-FABP

Serum or urine biomarkers offer an earlier prediction compared to serum creatinine; however, implementation remains limited due to a lack of standardized assay methodologies. This area is still under active research (51, 52). Serum NGAL, evaluated in a 2023 meta-analysis (five studies, 634 patients) indicated a sensitivity of 0.68, specificity of 0.88, and AUROC of 0.74 (27). Meanwhile, a 2024 prospective study (post–cardiopulmonary bypass) reported a higher sensitivity of 0.83, but a lower specificity of 0.64, with an AUROC of 0.67 (53). In contrast, urinary NGAL, assessed in a meta-analysis of 12 studies (1,391 participants) achieved a sensitivity of 0.75, specificity of 0.87, and an AUROC of 0.87 (27), although its clinical applicability of NGAL post cardiac surgery with CPB remains controversial (28, 54). Additional pooled data from 11 studies (1,541 participants, mostly PICU/post–cardiac surgery), revealed a sensitivity of 0.76, specificity of 0.77, and an AUROC of 0.77 for serum cystatin C (27), while urine IL-18, based on five studies (744 participants), yielded a sensitivity of 0.46, specificity of 0.78, and an AUROC of 0.76 (27). Moreover, the meta-analysis of four studies (585 patients) reported a an AUROC of 0.86 for the L-FABP, which is subsequently higher than serum cystatin, IL-8, and serum NGAL but comparable to urine NGAL (27).

### Plasma mRNA, miR-184, miR-6766-3p and combined

In a single-center pediatric study (20 patients), combining miR-184 and miR-6766-3p improved diagnostic accuracy (sensitivity 0.75, specificity 0.875), and accuracy of 0.8645 (Youden index: 0.625), indicating that a combined biomarker approach may enhance diagnostic performance in this setting (53).

### KIM-1, TIMP-2*IGFBP7, CHI3L1, VCAM, CCL14, and C-X-CL 10

Urine KIM-1 showed an AUROC of 0.72 in a pooled meta-analysis, while TIMP-2*IGFBP7 reached 0.77. Beta-2-microglobulin and serum IL-6 both averaged around 0.71–0.72 (27). In pediatric cardiac surgery, urine CHI3L1 and IGFBP7 had only moderate performance when not corrected for dilution, and serum creatinine change still performed better overall (55). Elevated VCAM levels were noted in AKI cases at six-hour intervals, whereas CCL14 appeared similar between AKI and non-AKI groups. CXCL10 rose significantly in AKI patients at 24–72 h post-bypass (56, 57).

### Limitations of this review

Most studies relied on robust internal and cross-validation across multiple cohorts or centers instead of external validation. Notably, two studies with large sample sizes may offer sufficient validation (36, 45), although one study cautioned that the absence of external validation combined with high-dimensional data could lead to overfitting in small cohorts.

### Recommendations

To validate the use of ML in predicting post-cardiac surgery-associated acute kidney injury (CS-AKI), future studies should be designed as prospective, multi-site trials (50). Additional research is needed to determine whether pretrained ML frameworks that incorporate dynamic, real-time temporal sequence, which provide continuous risk scores, can outperform models trained on static pre-collected data. Alternatively, deep networks employing multi-head attention, rather than single-head attention, might offer a more efficient processing and in-depth understanding of the data (58). Furthermore, greater emphasis should be placed on exploring and understanding the risk factors and associations of clinically significant AKI post-cardiac surgery in relation to mortality, and on evaluating whether pre-emptive detection and intervention in high-risk cases can reduce subsequent mortality.

While numerous investigations demonstrate strong predictive accuracy, translating these algorithms into clinical practice encounters significant barriers that current research fails to adequately examine (59). Live system deployment demands robust technological frameworks capable of handling complex computational demands, yet the incorporation of AI tools into existing clinical processes remains insufficiently studied (60). Though advanced warning systems and early alerts may facilitate timely interventions, their effectiveness depends on synchronization with relevant clinical workflows and actionable treatment windows (61). Zeng et al. (2023) noted that once trained, a model applies its fixed parameters to new patient data without needing to be retrained, thereby reducing computational demands and enabling real-time predictions (32). Similarly, ML data were used to develop a nomogram that lets clinicians calculate the one-year post-surgery AKD risk (35).

Economic evaluations examining implementation costs should be standard practice across machine learning healthcare applications, extending beyond CS-AKI research (62). These assessments must weigh the potential clinical advantages of early intervention against the financial burden of system deployment, including technology infrastructure, personnel education, and operational overhead (63). The absence of rigorous economic analysis leaves questions about the practical viability of integrating ML tools into clinical practice (64, 65).

Additionally, the research community should pivot from emphasizing predictive accuracy alone toward conducting real-world validation trials that reveal deployment obstacles (66). Future investigations need to examine practical applicability, healthcare system integration, and cost-effectiveness across varied institutional environments, rather than relying solely on statistical performance indicators (67).

## Conclusion

ML models predicting AKI in post-cardiac surgery pediatric patients demonstrate excellent discriminatory performance with prediction lead times up to two days, potentially outperforming traditional biomarkers and KDIGO criteria that often exhibit delayed presentations. These computational models appear superior to existing biomarkers and standard KDIGO classifications, which typically recognize kidney dysfunction only after significant damage has occurred. The strength of these algorithms lies in their capacity to synthesize complex perioperative data, from baseline patient characteristics through surgical variables like bypass time and perfusion adequacy to postoperative monitoring parameters. Despite these encouraging results, several critical concerns temper enthusiasm for immediate clinical adoption. The quality of available evidence suffers from significant methodological weaknesses, most notably the absence of rigorous external validation across different institutions and patient populations. This limitation is particularly troubling given the predominance of single-site retrospective studies, which create substantial risk for model overfitting and poor generalizability. Furthermore, the heterogeneity in study designs, patient selection criteria, and outcome measurements makes it difficult to draw definitive conclusions about true clinical effectiveness. A substantial gap persists between promising research outcomes and practical healthcare implementation. Current models largely rely on static data inputs rather than the continuous, dynamic monitoring that characterizes modern intensive care. Moving forward, the field requires well-designed prospective trials conducted across multiple centers with standardized protocols and outcome measures. Only through such rigorous validation can we determine whether these promising tools will truly enhance patient care in diverse clinical settings.

## Data Availability

The original contributions presented in the study are included in the article/Supplementary Material, further inquiries can be directed to the corresponding author.
