# Supplementary material for: The application of machine learning in predicting post-cardiac surgery acute kidney injury in pediatric patients: a systematic review
Source: Front Pediatr. 2025 Aug 12;13:1581578. doi: 10.3389/fped.2025.1581578 (PMC12378388; doi:10.3389/fped.2025.1581578)
Supplement: Supplementary file 1 [file Datasheet1.docx]

**Supplementary search**

Supplementary search strategy for PubMed S1

Pubmed Jun 27 2025: Results 14

Search: ("machine learning"[MeSH Terms] OR "artificial intelligence"[MeSH Terms] OR "machine learning"[tiab] OR "deep learning"[tiab] OR "neural network*"[tiab] OR "random forest"[tiab] OR "support vector machine"[tiab] OR "decision tree*"[tiab] OR "gradient boosting"[tiab] OR "XGBoost"[tiab] OR "classification model*"[tiab]) AND ("acute kidney injury"[MeSH Terms] OR "acute kidney injury"[tiab] OR "AKI"[tiab] OR "renal failure"[tiab] OR "renal dysfunction"[tiab]) AND ("cardiac surgical procedures"[MeSH Terms] OR "heart surgery"[tiab] OR "cardiac surgery"[tiab] OR "cardiopulmonary bypass"[tiab] OR "cardiothoracic surgery"[tiab]) AND ("pediatrics"[MeSH Terms] OR "infant"[MeSH Terms] OR "child"[MeSH Terms] OR "adolescent"[MeSH Terms] OR pediatric[tiab] OR paediatric[tiab] OR child*[tiab] OR infant*[tiab] OR neonate*[tiab])

Supplementary search strategy for Medline via Ovid with Embase S2

**Database:**
Your Journals@Ovid
Medline via ovid <1974 to 2025 Jun 27>

| **#** | **Query** | **Results from 24 Mar 2025** |
| --- | --- | --- |
| 1 | exp Machine Learning/ | 566,873 |
| 2 | exp Artificial Intelligence/ | 133,453 |
| 3 | (machine learning or artificial intelligence or deep learning or neural network* or random forest or support vector machine* or decision tree* or gradient boosting or XGBoost or classification model*).ti,ab. | 452,216 |
| 4 | 1 or 2 or 3 | 761,013 |
| 5 | exp Acute Kidney Injury/ | 140,645 |
| 6 | (acute kidney injury or AKI or renal failure or renal dysfunction).ti,ab. | 307,168 |
| 7 | 5 or 6 | 355,158 |
| 8 | exp Heart Surgery/ or exp Cardiopulmonary Bypass/ or exp Cardiac Surgical Procedures/ | 522,727 |
| 9 | (cardiac surgery or heart surgery or cardiopulmonary bypass or cardiothoracic surgery).ti,ab. | 174,746 |
| 10 | 8 or 9 | 587,807 |
| 11 | exp Pediatrics/ or exp Infant/ or exp Child/ or exp Adolescent/ | 4,291,113 |
| 12 | (pediatric or paediatric or child* or infant* or neonate*).ti,ab. | 3,887,844 |
| 13 | 11 or 12 | 5,940,805 |
| 14 | 4 and 7 and 10 and 13 | 27 |

Supplementary search strategy for Medline via Ovid with Embase S3

**Database:**
Web of Science
Jun 27 2025: Results 15

TS=(("machine learning" OR "artificial intelligence" OR "deep learning" OR "neural network*" OR "random forest" OR "support vector machine" OR "decision tree*" OR "gradient boosting" OR "XGBoost" OR "classification model*") AND ("acute kidney injury" OR "AKI" OR "renal failure" OR "renal dysfunction") AND ("cardiac surgical procedures" OR "heart surgery" OR "cardiac surgery" OR "cardiopulmonary bypass" OR "cardiothoracic surgery") AND (pediatric OR paediatric OR child* OR infant* OR neonate* OR adolescent*))

**Database:**
Scopus
Jun 27 2025: Results 9

#1 TITLE-ABS-KEY("machine learning" OR "artificial intelligence" OR "deep learning" OR "neural network*" OR "random forest" OR "support vector machine" OR "decision tree*" OR "gradient boosting" OR "XGBoost" OR "classification model*")

AND

#2 TITLE-ABS-KEY("acute kidney injury" OR "AKI" OR "renal failure" OR "renal dysfunction")

AND

#3 TITLE-ABS-KEY("cardiac surgical procedures" OR "heart surgery" OR "cardiac surgery" OR "cardiopulmonary bypass" OR "cardiothoracic surgery")

AND

#4 TITLE-ABS-KEY(pediatric OR paediatric OR child* OR infant* OR neonate* OR adolescent*)
