# Supplementary material for: The application of machine learning in predicting post-cardiac surgery acute kidney injury in pediatric patients: a systematic review
Source: Front Pediatr. 2025 Aug 12;13:1581578. doi: 10.3389/fped.2025.1581578 (PMC12378388; doi:10.3389/fped.2025.1581578)
Supplement: Supplementary file 2 [file Table1.docx]

Supplementary table 1

| **Author (Year)** | **Limitations** | **Recommendations** |
| --- | --- | --- |
| Dong (2021) $ | 1. AKI diagnosis limited to creatinine, excludes urine output criteria;  2. US dataset dominance skews validation. | 1. Conduct further real-world testing and integration into clinical workflows.  2. Expand predictors to include urine output data for improved accuracy. |
| Fragasso (2023) | 1. Retrospective study design: Limits causal inference and may introduce bias.  2. Small sample size: May not represent the full variability of pediatric cardiac ICU populations.  3. Excluded risk factors: Missing key AKI predictors, such as antibiotic use, surgical risk scores, and cardiac anatomy.  4. External validation: Lack of testing in other PCICUs or on new datasets limits generalizability.  5. Transient vs. persistent AKI: Does not distinguish between transient and persistent cases, affecting clinical insights.  6. Clinician usability: ML models, like RF, may be complex for bedside application without proper training or integration.  7. Data handling: Missing data and imputation techniques might influence predictions.  8. Urine output challenges: Difficulty in integrating accurate urine output data impacts prediction reliability. | 1. Prospective Validation: Conduct multicenter prospective studies to confirm model performance and generalizability.  2. Inclusion of Key Variables: Add clinical factors like nephrotoxic drug use, surgical risk scores, and cardiac anatomy.  3. Simplify Clinical Integration: Develop user-friendly interfaces or dashboards for easy application by clinicians.  4. Differentiate AKI Types: Enhance the model to distinguish between transient and persistent AKI.  5. Improve Explainability: Apply SHAP or LIME to increase model interpretability and clinician trust.  6. Expand Sample Size: Increase cohort size to boost statistical power and address population variability.  7. Develop Preventive Bundles: Create tailored interventions for patients at risk of AKI.  8. Integrate Novel Biomarkers: Explore biomarkers like NGAL or cystatin C for better prediction accuracy. |
| Luo (2023) | 1. Retrospective design: Relies on historical data, introducing bias and limiting causal inference.  2. Single region: Data from Chinese tertiary institutions may restrict generalizability to other regions or healthcare settings.  3. Urine output data unavailable: Excluded from CS-AKI definition, potentially underestimating AKI incidence.  4. Missing data: Imputation methods may affect model accuracy.  5. No validation in diverse populations: Validation was limited to two centers in the same region.  6. Causality unclear: No evidence that modifying predictors reduces CS-AKI risk.  7. Model complexity: Algorithms like XGBoost may hinder real-time clinical adoption without interpretability tools.  8. Potential overfitting: Small subgroups may risk overfitting despite cross-validation.  9. Limited modifiable predictors: Many predictors, such as perfusion time and baseline creatinine, are difficult to modify. | 1. Prospective Studies: Conduct prospective multicenter studies to enhance generalizability and minimize bias.  2. Incorporate Diverse Populations: Validate models across varied geographic and demographic settings.  3. Include Urine Output Criteria: Integrate urine output data with serum creatinine for improved AKI definitions.  4. Simplify Model Outputs: Create user-friendly interfaces or risk calculators for real-time clinical use.  5. Explore Interventions: Test whether modifying predictors (e.g., perfusion time) reduces CS-AKI risk in randomized trials.  6. External Tool Integration: Embed the model into electronic health records for real-time predictions and clinician alerts.  7. Focus on Modifiable Predictors: Investigate perioperative predictors that can be adjusted to lower AKI risk.  8. Additional Biomarkers: Incorporate novel biomarkers to boost model performance.  9. Implementation Science: Assess the impact on clinical workflows, patient outcomes, and cost-effectiveness. |
| Zeng (2023) | 1. Single-center data: Conducted at a single institution (Children’s Hospital, Zhejiang University), limiting generalizability to populations with differing demographics, practices, or healthcare systems.  2. AKI definition based solely on serum creatinine: Excluded urine output criteria, potentially missing AKI cases and underestimating incidence, which could bias model performance.  3. Model complexity and interpretability: More complex than simpler models like logistic regression, despite SHAP interpretation, potentially hindering clinical adoption due to reliance on "black box" methods.  4. Imbalanced data: Addressed with oversampling (e.g., SMOTE), which may not accurately reflect real-world prevalence and could overestimate performance in rare-event predictions.  5. Time and resource intensive: Requires significant computational resources, making implementation challenging for smaller or low-resource healthcare facilities.  6. Unexplored factors: Socioeconomic variables, genetic predispositions, and hospital resource variability were not analyzed, potentially overlooking key influences on AKI outcomes.  7. External validation missing: Lack of testing on datasets from other centers or populations limits the model’s robustness and transferability. | 1. External Validation: Validate the model with multicenter datasets across diverse populations to ensure robustness in varied clinical settings.  2. Incorporate Additional AKI Criteria: Include urine output criteria and novel biomarkers like NGAL and cystatin C to improve AKI detection and model performance.  3. Simplify the Model: Develop interpretable versions using hybrid approaches, blending traditional and deep learning methods to enhance clinical usability.  4. Address Data Imbalance: Apply advanced techniques, such as cost-sensitive learning or weighted loss functions, to better handle imbalanced datasets in real-world scenarios.  5. Real-Time Integration: Integrate the model into electronic health record systems for real-time predictions and decision support.  6. Broader Feature Set: Expand features to include social determinants of health, genetic factors, and institutional variables influencing AKI risk.  7. Cost-Benefit Analysis: Conduct cost-effectiveness studies to evaluate the model’s feasibility, particularly in resource-limited settings.  8. Prospective Validation: Test the model in prospective clinical trials to assess its impact on real-world outcomes and decision-making.  9. Engage Stakeholders: Collaborate with clinicians, data scientists, and administrators to design user-friendly interfaces and workflows for routine care adoption.  10. Dynamic Updates: Implement a system for periodic updates to ensure the model remains accurate with evolving data, technologies, and clinical practices. |
| Hayward (2023) | 1. Single-center retrospective design: Data from a single institution limits generalizability to other settings, and the retrospective nature prevents causal inference, with potential inconsistencies or missing data.   2. Short timeframe for AKI diagnosis: AKI assessment within 48 hours may have missed late-onset or more severe cases, underestimating the impact of perioperative factors on kidney injury.   3. Lack of granular data on medications and practices: Key variables, such as vasoconstrictor use during CPB and individual surgeon or perfusionist practices, were not collected, potentially overlooking important influences on AKI development.   4. Heterogeneity in AKI definition: Using the KDIGO criteria, while common, introduces interstudy variability due to differing pediatric AKI definitions. Serum creatinine-based classification is also affected by factors like age, muscle mass, and nutrition, risking misclassification.   5. Exclusion criteria: Excluding patients with circulatory arrest or hypothermia ensures a homogeneous sample but reduces applicability to those undergoing complex procedures.   6. Random Forest model limitations: Modest predictive accuracy (AUC: 0.67) suggests the model does not fully capture AKI complexity, and its improvement over logistic regression was not statistically significant (p = 0.294).   7. Small subgroup analyses: Limited analysis of subgroups, such as patients with cyanotic heart disease, restricts understanding of specific risk profiles. | 1. Prospective Validation: Conduct multicenter prospective studies to improve generalizability and assess causal relationships.  2. Broader Timeframe for AKI Assessment: Extend follow-up periods to capture late-onset AKI and evaluate long-term renal outcomes.  3. Incorporation of Additional Variables: Collect detailed intraoperative data, including vasopressor use and perfusionist-specific techniques, to explore their impact on AKI risk.  4. Exploration of Patient-Specific Management: Investigate individualized oxygen delivery goals for subgroups like cyanotic patients who may tolerate lower DO2i levels.  5. Development of Improved Predictive Models: Utilize advanced machine learning techniques or ensemble models and incorporate early detection biomarkers to enhance predictive accuracy.  6. Refinement of AKI Definitions: Establish consensus on pediatric-specific AKI definitions for consistency in research.  7. Focus on DO2i Threshold Management: Perform interventional studies to evaluate the effectiveness of maintaining DO2i >350 mL/min/m² in reducing AKI incidence.  8. Investigation of Hyperoxia Risks: Study the risks associated with prolonged hyperoxia, particularly in cyanotic patients, to define safe upper oxygen delivery limits. |
| Nagy (2024) | 1. Sample Size and Generalizability:  Small cohort size for machine learning (ML) analysis. Data limited to a single-center experience, reducing generalizability.  2. Incomplete Physiologic and Treatment Data:  Missing key intraoperative data, such as hypotension episodes, urine output, transfusions, and vasopressor use.  3. Temporal Data Gap:  Data collected between 2007 and 2013 may not fully reflect modern practices, despite minimal changes in surgical techniques.  4. Limited Data Collection Frequency:  Manual data retrieval limited the ability to capture more frequent assessments or dynamic changes.  5. Excluded Biomarkers:  Lack of advanced biomarkers (e.g., NGAL, cystatin C) and measures like eGFR or fluid overload that could enhance prediction accuracy.  6. Endpoint Limitations:  Use of KDIGO stage 2 or 3 criteria for CS-AKI may not fully address clinical utility or represent broader AKI phenotypes.  7. Limited Prediction for KRT:  Few cases of kidney replacement therapy (KRT) in the dataset made the model unsuitable for this outcome. | 1. Expand Sample Size and Validation: Conduct multicenter studies with larger datasets to improve generalizability and include external validation cohorts.  2. Enhance Data Completeness: Gather detailed intraoperative and postoperative physiological and treatment variables.  3. Update Data Sources: Utilize contemporary datasets to ensure alignment with current clinical practices.  4. Increase Data Granularity: Automate data collection for frequent and detailed sampling of clinical variables.  5. Incorporate Advanced Biomarkers: Add NGAL, cystatin C, eGFR, and fluid overload as predictive features.  6. Refine Endpoints: Consider alternative or composite endpoints to capture diverse CS-AKI phenotypes.  7. Specialized KRT Models: Develop models specifically tailored for predicting KRT outcomes with sufficient data.  8. Prospective Design: Employ prospective data collectors to reduce bias and maintain up-to-date insights. |
| Loomba (2024) | 1. Small sample size: Inclusion of only 9 patients limits generalizability and reduces study power despite over 27,000 datapoints.  2. Single-center design: Findings may reflect site-specific practices, limiting broader applicability.  3. Retrospective nature: Prevents establishing causal relationships between variables and AKI.  Lack of external validation: The risk score remains untested in independent cohorts, restricting confirmation of predictive accuracy.  4. Limited variables: Focused on hemodynamic parameters, excluding significant factors like nephrotoxic medications and kidney biomarkers.  5. Exclusion of urine output: AKI definition relied solely on serum creatinine, omitting a key diagnostic criterion.  6. Temporal resolution vs. causality: High-resolution data cannot overcome the causal limitations of retrospective design.  7. Simplified vasoactive inotropic score: Does not differentiate between specific vasoactive medications or their renal effects. | 1. Expand Sample Size and Multicenter Collaboration: Conduct larger, multicenter studies to validate findings and improve generalizability across institutions and patient populations.  2. Include Broader Patient Demographics: Test the model's applicability in diverse cohorts with varying cardiac conditions and surgical techniques.  3. Prospective Validation: Design prospective studies to evaluate the AKI risk score’s predictive power in real-time clinical settings.  4. External Validation: Compare the risk score against existing AKI prediction models or benchmarks to assess performance and utility.  5. Incorporate Additional Variables: Add predictors like biomarkers (e.g., NGAL, cystatin C), medications, and comprehensive clinical parameters.  6. Urine Output Monitoring: Include urine output criteria to create a more comprehensive AKI assessment.  7. Investigate Interventions: Study how using the risk score in real-time influences clinical decisions, such as fluid management or vasoactive use.  8. Refinement of Machine Learning Models: Use advanced techniques like random forests and neural networks to enhance model accuracy, especially for smaller sample sizes.  9. Longitudinal Follow-up: Examine long-term renal outcomes in AKI patients, stratified by identified risk factors. |
| Kong (2023) | 1. Single-center retrospective design with a modest sample size, which may limit generalizability. 2. Absence of external validation raises concerns about overfitting and the model’s stability in other settings. 3. Lack of incorporation of additional lower body perfusion techniques that could influence renal outcomes. | 1. . Conduct multicenter studies with larger, prospectively collected datasets to externally validate and refine the model. 2. Consider including additional predictors or imaging biomarkers to further enhance predictive performance and clinical applicability. |
| Tong (2024) | 1. Single-center, retrospective design with potential unmeasured confounding. 2. Limited perioperative data granularity; important variables such as blood transfusion, vasopressor dosage, opioid use, and surgeon experience were not included. 3. Absence of subgroup analysis by specific surgical types. 4. Model interpretation methods (e.g., SHAP) may not fully account for feature dependencies. 5. No external validation, limiting generalizability. | 1. Incorporate additional perioperative variables in future analyses. 2. Conduct subgroup analyses based on different surgical types. 3. Validate the model in multicenter, prospective cohorts to enhance generalizability. 4. Explore improved interpretability techniques that account for correlations among predictors |
